# Phylogeny of Bacterial and Archaeal Genomes Using Conserved Genes: Supertrees and Supermatrices

**DOI:** 10.1371/journal.pone.0062510

**Published:** 2013-04-25

**Authors:** Jenna Morgan Lang, Aaron E. Darling, Jonathan A. Eisen

**Affiliations:** 1 Department of Medical Microbiology and Immunology and Department of Evolution and Ecology, University of California Davis, Davis, California, United States of America; 2 Department of Energy Joint Genome Institute, Walnut Creek, California, United States of America; Columbia University, United States of America

## Abstract

Over 3000 microbial (bacterial and archaeal) genomes have been made publically available to date, providing an unprecedented opportunity to examine evolutionary genomic trends and offering valuable reference data for a variety of other studies such as metagenomics. The utility of these genome sequences is greatly enhanced when we have an understanding of how they are phylogenetically related to each other. Therefore, we here describe our efforts to reconstruct the phylogeny of all available bacterial and archaeal genomes. We identified 24, single-copy, ubiquitous genes suitable for this phylogenetic analysis. We used two approaches to combine the data for the 24 genes. First, we concatenated alignments of all genes into a single alignment from which a Maximum Likelihood (ML) tree was inferred using RAxML. Second, we used a relatively new approach to combining gene data, Bayesian Concordance Analysis (BCA), as implemented in the BUCKy software, in which the results of 24 single-gene phylogenetic analyses are used to generate a “primary concordance” tree. A comparison of the concatenated ML tree and the primary concordance (BUCKy) tree reveals that the two approaches give similar results, relative to a phylogenetic tree inferred from the 16S rRNA gene. After comparing the results and the methods used, we conclude that the current best approach for generating a single phylogenetic tree, suitable for use as a reference phylogeny for comparative analyses, is to perform a maximum likelihood analysis of a concatenated alignment of conserved, single-copy genes.

## Introduction/Background

Until relatively recently, the evolution of species was largely assumed to be a strictly bifurcating, tree-like process [1]–[3]. However, many argue that such a tree-like depiction of the history of species, especially for microbes, is not valid because the high incidence of movement of genes from one lineage to another (i.e., horizontal gene transfer, or HGT) is thought to obscure a vertical line of descent [4]–[7]. Though unquestionably HGT is of great importance in understanding the biology and evolution of species, many studies have found that some genes appear to be transferred horizontally at a much lower rate than others [8]. Such genes can thus potentially be used to reconstruct a framework of vertical inheritance of microbial species [9], [10]. Such a framework is important, because even though it might not perfectly reflect the evolutionary history of every gene within a given organism, or even of each organism, it is useful as a first approximation to the evolutionary history of organisms [11]. For example, it is from within this framework that events like horizontal gene transfer can be detected and understood as significant deviations from a first approximation. Also, phylogenetic trees are useful tools for many other applications. They can be used for guiding the selection of genomes for sequencing [12]; assigning taxonomy to community (metagenomic and 16S PCR) sequence data [13], [14]; identifying ecological trends [14]–[17]; inferring co-speciation [18], [19], epidemiological [20]–[22], and biogeographical [23], [24] events; performing phylogenetic profiling analysis [25], and more.

More than 3000 bacterial and archaeal genomes have been sequenced and deposited in public databases to date, including the results of a large-scale effort to choose organisms for genome sequencing based on their phylogenetic diversity [12]. The phylogenetic relationships among many of the sequenced genomes are unclear. When new species are described, it is commonplace to use a phylogeny of the gene for the small-subunit ribosomal RNA (in bacteria and archaea, this is known as the 16S rRNA gene, in eukaryotes, it is known as the 18S rRNA gene) to place them in a phylogenetic context. The 16S rRNA gene is a valuable tool for this purpose because its sequence has regions of both low and high conservation and because there are now hundreds of thousands of sequences available from both cultured and environmental organisms. However, it is likely that there will be differences between a phylogenetic tree inferred using the 16S rRNA gene versus other phylogenetic marker genes [26]. This is generally the case when comparing phylogenies reconstructed from different genes, because they may have different amounts of phylogenetic signal, evolutionary histories, or rates of evolution, and because issues like convergence, long-branch attraction, and hidden paralogy can lead to incorrect tree inference [1], [27].

This work is motivated by the need for a single, fully resolved, best estimate of the phylogeny of bacteria and archaea that will be used to perform various comparative analyses in a phylogenetic context. Here, we employ several phylogenetic methods to infer the phylogeny of the bacteria and archaea for which genome sequences are available, using a set of 24 single-copy, ubiquitous genes (Table 1). Alignments generated for these genes can either be concatenated for a single analysis (a *supermatrix* method), or used individually to infer a tree for each gene and then combining the gene trees to build a single organismal phylogeny (*supertree* and simultaneous gene tree/species tree methods). Both approaches have trade-offs and shortcomings that have been discussed extensively elsewhere [28]–[33]. To summarize, the primary drawback of the supermatrix approach is that a shared evolutionary history is assumed for all genes, and when this assumption is violated, inaccurate trees with strong measures of support can be obtained in some cases [33]. With supertree methods, important information is often lost during the construction of a supertree, including branch lengths and statistical measures of support for individual clades, and the information content of a single gene may not be sufficient to recover some relationships among organisms [34].

**Table 1:** Genes used in this study.

gene ID	gene name	gene product	length	avg % identity	avg RF	bootstrap reps
PMPROK00003	rplN	50S ribosomal protein L14	118	39%	1124.18	500
PMPROK00015	rpsC	30S ribosomal protein S3	180	46%	947.92	400
PMPROK00019	rpsE	30S ribosomal protein S5	155	47%	1026.01	500
PMPROK00020	rplF	50S ribosomal protein L6	168	49%	1039.08	450
PMPROK00025	rpsS	30S ribosomal protein S19	91	50%	1194.08	550
PMPROK00028	rpsB	30S ribosomal protein S2	226	51%	903.61	450
PMPROK00029	rplK	50S ribosomal protein L11	141	51%	1112.34	450
PMPROK00034	rplD	50S ribosomal protein L4	196	52%	947.99	450
PMPROK00041	rpsQ	30S ribosomal protein S17	78	52%	1222.31	450
PMPROK00048	rplB	50S ribosomal protein L2	208	52%	1015.17	500
PMPROK00051	rpsI	30S ribosomal protein S9	128	53%	1098.84	450
PMPROK00053	rplE	50S ribosomal protein L5	176	53%	1027.25	500
PMPROK00054	rpsG	30S ribosomal protein S7	156	54%	1011.56	450
PMPROK00060	lepA	GTP-binding protein LepA	598	56%	666.34	300
PMPROK00064	infB	translation initiation factor IF-2	533	56%	701.37	300
PMPROK00068	rpsK	30S ribosomal protein S11	113	58%	1149.50	500
PMPROK00071	rplP	50S ribosomal protein L16	133	58%	1070.93	450
PMPROK00074	rpsH	30S ribosomal protein S8	125	59%	1083.00	450
PMPROK00075	rplC	50S ribosomal protein L3	202	60%	950.51	400
PMPROK00081	rpsM	30S ribosomal protein S13	118	61%	1129.42	450
PMPROK00087	pheS	phenylalanyl-tRNA synthetase, alpha subunit	219	61%	920.66	450
PMPROK00092	rplO	50S ribosomal protein L15	138	62%	1128.83	400
PMPROK00093	rpsJ	30S ribosomal protein S10	101	65%	1094.34	400
PMPROK00094	rpsL	30S ribosomal protein S12	118	74%	1164.01	450

Data for the 24 genes used in this study, including the gene product, alignment length, average Robinson-Foulds (RF) distance among bootstrap replicates, the number of rapid bootstrap replicates required to reach convergence, and the amino acid model with the highest Bayesian posterior probability.

Previous work on large-scale microbial phylogenetics has primarily relied upon the supermatrix approach [35]–[37], but see also [38]–[40], We desired to explore the use of a new supertree approach (discussed below) that has not yet been applied to a dataset of this scale. We present the results of both concatenated alignment supermatrix and single-gene supertree approaches. In addition to the two different approaches to combining the information from several genes, we employed several methods of phylogenetic inference. We used RAxML to produce maximum likelihood trees and we used MrBayes to produce Bayesian trees. We attempted to use both RAxML and MrBayes for supermatrix and supertree analyses.

Many factors can contribute to the incongruence of phylogenetic trees inferred from different genes. In bacteria and archaea, horizontal gene transfer is the most common explanation offered in the literature, but incomplete lineage sorting, model violation, convergence, long branch attraction, and lack of phylogenetic signal can also contribute to phylogenetic incongruence. Several methods, including *BEAST [41], BEST [42], STEM [43], and BUCKy [44], have been developed to estimate an organismal phylogeny, given a collection of gene trees, the topologies of which may not be known with certainty. MRP (Matrix Representation Parsimony) [45], [46] requires the input of a single tree for each gene, a requirement we sought to avoid because we expected that the high sequence divergence:sequence length ratio might result in poorly resolved gene trees. MRP does not perform well in the presence of uncertainty or discordance [47]. Most of these methods were developed by researchers interested in plant and animal phylogenetics, where the number of taxa are few (relative to the number presented here), horizontal gene transfer is rare, and gene tree incongruence is more likely caused by variable rates of evolution, hidden paralogy, and incomplete lineage sorting. BUCKy is the only currently available method that is agnostic with respect to the cause of incongruence among gene trees. BUCKy implements Bayesian Concordance Analysis. It takes into account the uncertainty within an individual gene tree, does not assume that all genes share an evolutionary history, and provides a statistical means by which a “dominant” topology, called a primary concordance tree, can be obtained. Here, we employed BUCKy to calculate the primary concordance tree given the gene trees for the 24 single-copy, universally-distributed genes.

## Methods

### Taxa and Marker Selection and Alignment

PhyloSift (manuscript in preparation, software available at https://github.com/gjospin/PhyloSift) was run on the database of all bacterial and archaeal draft and complete genomes available from NCBI and IMG as of April 2011. PhyloSift (which is based partly on AMPHORA [13]) performs a blastx [48] search of genome nucleotide data and uses profile HMMs, built from high-quality, manually curated sequence alignments, to align sequence data for a set of phylogenetic marker genes. With PhyloSift, the set of marker genes includes 38 genes that are mostly single-copy and ubiquitous in the bacteria and archaea (Wu *et. al.,* manuscript in preparation). A multiple sequence alignment was generated for each of the 38 genes with hmmalign [49] by aligning each database hit to the profile HMM alignment. Only the 2966 organisms that had at least 18 of the 38 markers were used for further analyses.

In order to successfully complete some of the more computationally intensive phylogenetic analyses, we found it useful to remove all but one representative of sets of very closely related taxa. These redundant taxa contribute a great deal of uncertainty to the analysis near the tips of the phylogeny (because the phylogenetic marker genes are very highly conserved at the amino acid level, which is the level being analyzed here,) without contributing useful information to the inference of relationships among the closely-related species. Therefore, a subset of taxa was chosen that is representative of the total organismal diversity. In order to do this, we first built a phylogeny using FastTree [50] with the default settings (see Figure S3 and Datafile S3). This tree was used to guide an organism selection process based on the PD, or phylogenetic diversity (branch length), that each organism contributed to the tree. We applied the greedy max PD algorithm of Steel, 2005 [51], stopping when new organisms were contributing less than 2 substitutions per 100 sites. This resulted in the inclusion of 800 taxa.

In addition to eliminating redundant taxa, a related concern was one of minimizing the amount of missing data. Missing data are particularly problematic for the implementation of BUCKy because it requires that each taxon is present in every single-gene analysis. In order to apply BUCKy to data where a taxon is missing a gene, phylogenetic analysis for that gene must include that taxon with all sites coded as missing data. If many taxa are missing data, the result is a lot of uncertainty, causing a diffuse posterior distribution of topologies and inaccurate results [52]. So, an implementation [53] of an algorithm [54] that enumerates all maximal bicliques was used to find the set of markers that were present in all 800 taxa. This resulted in the selection of the 24 genes used for all analyses.

An additional 41 taxa were manually added to the initial list of 800 genomes. These additional genomes were either publicly unavailable (not yet deposited into Genbank) or of particular interest (because they represent under-sampled lineages) and missing one or more of the 24 marker genes. See Table S1 for a complete list of organisms used for this study. A few of these additional taxa were missing up to 14 marker genes, but no single marker gene was missing data for more than 6 taxa. This limits the degree of topological uncertainty that may be present in any gene tree due to missing data effects.

### Bayesian Inference of Phylogenies Using a Concatenated Alignment

In order to ensure that the results from the supermatrix and supertree approaches would be directly comparable, we used a concatenated alignment of the same 24 marker genes with the same 841 taxa as input for a Bayesian phylogenetic inference with MrBayes v. 3.2.0 [55]. The alignment was partitioned by gene, with the topology and branch lengths linked across partitions, but the amino acid model and shape parameter of the gamma distribution unlinked. Each partition was permitted to jump between 10 fixed amino acid models and the gamma distribution was approximated using 4 rate categories. The MCMC was run with a temperature parameter of 0.2, sample frequency of 100, swap frequency of 3, and 2 independent runs with 4 chains each for 1000000 generations. Convergence was assessed using the reported average standard deviation of split frequencies (ASDSF), which is a measure of the difference among the tree samples obtained in the different chains. The suggested value of this statistic, when the runs converge upon a solution, should fall below 0.01. All analyses presented here were run on a 1.6 GHz Intel Xeon CPU.

### Bayesian Inference of Phylogenies Using Single-gene Alignments

Alignments of the 24 marker genes selected to minimize the amount of missing data (as described above) were analyzed with MrBayes using the parameters above, for 1 million generations, requiring approximately 9 months of CPU time for each marker.

### Maximum Likelihood Inference of Phylogenies Using a Concatenated Alignment

A concatenated alignment of 24 genes for 841 taxa was used as input for maximum likelihood inference with RAxML v 7.2.8 [56]. The alignment was partitioned by gene, and ProtTest [57] was used to select the appropriate model of amino acid substitution for each partition. We used the –f a option of RAxML to generate 100 rapid bootstrap replicates [58] followed by a search for the best-scoring ML tree. We then assessed bootstrap convergence using the –I autoMRE option in RAxML and found that convergence had not been achieved. We then initiated a new rapid bootstrapping run, which was terminated upon convergence at 250 replicates (after 360 CPU days.).

### Maximum Likelihood Inference of Phylogenies Using Single-gene Alignments

Alignments of each of the 24 markers that were selected to minimize the amount of missing data were analyzed with RAxML as described above for the concatenated alignment. However, because some taxa were composed entirely of missing data for some of the single-gene analyses, the RAxML source code was modified to permit the analysis to run with these taxa included. We used the –f a option of RAxML to generate 100 rapid bootstrap replicates [58] followed by a search for the best-scoring ML tree for each gene. We then assessed bootstrap convergence using the –I autoMRE option in RAxML and found that convergence had not been achieved. We then initiated a new rapid bootstrapping run to generate 1000 replicates. Convergence was assessed using the –I autoMRE option in RAxML. A majority-rule consensus tree was calculated using the 24 best ML trees produced by RAxML, using the –J MR option in RAxML.

### 16S rRNA Gene Phylogeny

16S rRNA sequences for each of 841 organisms were either downloaded from NCBI or retrieved manually from the IMG database [59]. For the cases in which multiple copies of the 16S rRNA gene were present in a single genome, the longest sequence was selected for further analysis. They were aligned using Infernal using a covarion model built from a high-quality reference alignment [60]. RAxML was used with the –f a option and the GTR+Γ model of nucleotide substitution to generate 1000 bootstrap replicates followed by a search for the best-scoring ML tree.

### BUCKy

For reasons discussed below in the results, we did not use BUCKy with the results of the single-gene Bayesian phylogenetic analyses. Instead, we used BUCKy to generate a primary concordance tree (referred to hereafter as the “BUCKy” tree) from the single-gene RAxML bootstrap replicates. To convert RAxML output into a format acceptable as input for BUCKy, a custom perl script, dependent on R, is provided in Datafile S7. In order to reduce the memory required to run BUCKy to a level acceptable by currently available computational resources, the population tree was not computed. In order to modify the code to remove this functionality, the line:

bool buildPopulationTree = false;

was added immediately after the line:

string quartetTree, quartetTreeWithWts;

We evaluated the effect of choosing different prior values for the alpha parameter on the results generated by BUCKy. We ran BUCKy using the default prior of 1, which for our data, centers the prior distribution on the number of distinct gene trees around 3.5. We also used alphas of 10, 50, and 100, for which the prior distribution is centered around 12.5, 18, and 22 trees, respectively.

### Tree Comparison and Visualization

We used the Robinson-Foulds (RF) metric [61] to quantify the distance between the concatenated ML tree topology and the BUCKy tree topology, and between each of them and the 16S rRNA tree topology. The treedist algorithm available in the PHYLIP software package [62] was used to compute the symmetric distance (RF) between pairs of trees. While this metric provides a means by which to state that one tree is more similar to a second tree than it is to a third tree, given our data, there is no means by which to state that two trees are significantly different from each other at a given probability level. Dendroscope [63] was used to visualize and annotate single phylogenetic trees and to generate the majority-rule consensus tree.

## Results and Discussion

### Taxa/markers Used for Analysis

In large part because not every genome used in this analysis was completely finished, if we had restricted our analysis to genes that were universally present in all the genomes analyzed, we would have been left with only four ribosomal proteins. Given the amount of total phylogenetic diversity among these organisms, a phylogeny reconstructed using only four ribosomal protein (i.e. short) genes would certainly be too poorly resolved for our purposes. We did attempt to minimize the impact of missing genes by limiting the number of missing sequences per gene to no more than six.

841 organisms, including four plastid and three mitochondrial genomes and a subset of 24 of the PhyloSift phylogenetic markers were used for all initial analyses. In an attempt to minimize artifacts due to long-branch attraction (discussed below) at the split between the bacteria and archaea, we also built trees from bacteria-only alignments of 761 taxa. See Table 1 and Table S1 for a list of genes and organisms used, respectively.

The total phylogenetic diversity (branch length) contained in a tree of all 16S rRNA sequences maintained in the Greengenes database [64] is 1766.27. A comparable tree of our subsample of 841 organisms (bacteria+archaea) has a total branch length of 69.91. Therefore, our current phylogenetic analysis includes approximately 4% of the known phylogenetic diversity found in the Greengenes database [65].

### Long Branch Attraction (LBA)

Long branch attraction (LBA) is a well-known phylogenetic artifact that causes sequences that are on long branches (which can occur because the lineages have accelerated evolutionary rates or because they are on isolated evolutionary branches) to incorrectly appear to be closely related [66]. This artifact was originally thought to mainly affect maximum parisomy analyses, but recent simulation studies have shown that, even when the correct model of evolution is selected, maximum likelihood analyses are not immune [67]. LBA can also cause organisms on long branches to be pulled to the root of the tree (i.e., toward the outgroup) [68]. Therefore, when interpreting phylogenetic trees, it is important to be particularly skeptical about the relationships of organisms that are on long branches, especially when they appear to be sister taxa or branch deeply in a rooted tree. The concatenated ML tree, using the archaea as an outgroup to the bacteria, displays several instances of likely LBA (Figure S4 and Datafile S4). In particular, our tree has six suspicious bacterial branches that lead to candidate division TM7 single cell_isolate TM7c, candidate division TM7 genomosp GTL1, *Mycoplasma suis*, Candidatus *Carsonella ruddii*, and Candidatus *Hodgkinia cicadicola*. These organisms are either missing up to 10 of the marker genes (because they are intracellular symbionts with streamlined genomes or single-cell isolates with incomplete genomes) or have accelerated rates of sequence evolution, or both.

One way to reduce the impact of LBA, in terms of drawing long branches to the root, is to remove the distantly-related outgroup taxa. We repeated the RAxML analysis with all of the archaeal species removed from the concatenated alignment. When we did this, each of these six long branches moved to different locations in the concatenated ML tree. The two *Mycoplasma* species moved, with high bootstrap support (100%) to a clade with the other *Mycoplasma* species. The two insect endosymbionts, *Hodgkinia* and *Carsonella*, moved to within the Proteobacteria, but there they form a poorly-supported clade with another long-branch taxon (candidate division TM7 genomosp. GTL1), suggesting that LBA may still be an issue with the placement of these taxa. The two TM7 genomes were moved to different locations in the tree, as discussed in the TM7 section. In the BUCKy tree with only bacterial taxa, the *Mycoplasma* species moved to the *Mycoplasma* clade; the two TM7 genomes do have a sister relationship, and they form a lineage basal to the Actinobacteria; *Hodgkinia* becomes the basal lineage of the alpha-proteobacterial clade; and *Carsonella* is sister to Candidatus *Zinderia insecticola*, a beta-proteobacterial insect endosymbiont. *Carsonella+Zinderia* form a clade with the mitochondrial genomes, and that clade is sister to the alpha+beta-proteobacterial clade.

Aside from the TM7 genomes, which have never been phylogenetically placed with confidence, each of these moves were to positions that were at least closer to expectation given the 16S rRNA tree and other, independent, physiological lines of evidence [69]–[71]. It is likely that the bacteria-only trees may still suffer from artifacts of phylogenetic reconstruction (LBA or others), but because we assume that they represent a better estimate of the bacterial phylogeny than the trees that include the archaea, we will use bacteria-only trees for all further analyses and tree comparisons. We note that in the trees presented here of bacteria+archaea, the relationships among the archaea are congruent with recently published phylogenies of the archaea, including a ML tree based on a concatenated alignment of 57 ribosomal proteins [72] that shows higher clade support values than the concatenated ML tree presented here.

### Bayesian Phylogenetic Analyses

For both the concatenated alignment and for all single-gene alignments, after approximately 18 months of CPU time, two million MCMC generations had completed in MrBayes. However, the average standard deviation of split frequencies (ASDSF) for all runs was > = 0.16, which is well above the level (0.01) suggested by the authors of the software to be indicative of convergence. And, in majority-rule consensus trees, generated after discarding the first 25% of the trees sampled as burn-in, not a single node had greater than the 0.5 posterior probability required for inclusion in a majority rule consensus tree.

Because it is impossible to know if or when these analyses would ever converge upon an answer, given our computational resources, we opted to abandon them. Recent work in Bayesian phylogenetics has demonstrated new algorithms that can run several orders of magnitude faster than MCMC on large datasets like ours [73], but these are not currently available in high-quality implementations.

### Maximum Likelihood Phylogenetic Analyses

We concluded that the best approaches to reconstructing the phylogeny of the bacteria and archaea, given our time limits and computational resources were by 1) running a Maximum Likelihood search using RAxML on a partitioned, concatenated alignment of the 24 single-copy phylogenetic marker genes and 2) using bootstrap replicates for each of the 24 phylogenetic marker genes as input for a Bayesian Concordance Analysis using BUCKy to produce a primary concordance tree [40].

We performed 1000 bootstrap replicates for each gene, and assessed convergence using the –I autoMRE option in RAxML. Bootstrapping converged for all genes after no more than 500 replicates (Table 1).

**Figure 1:**
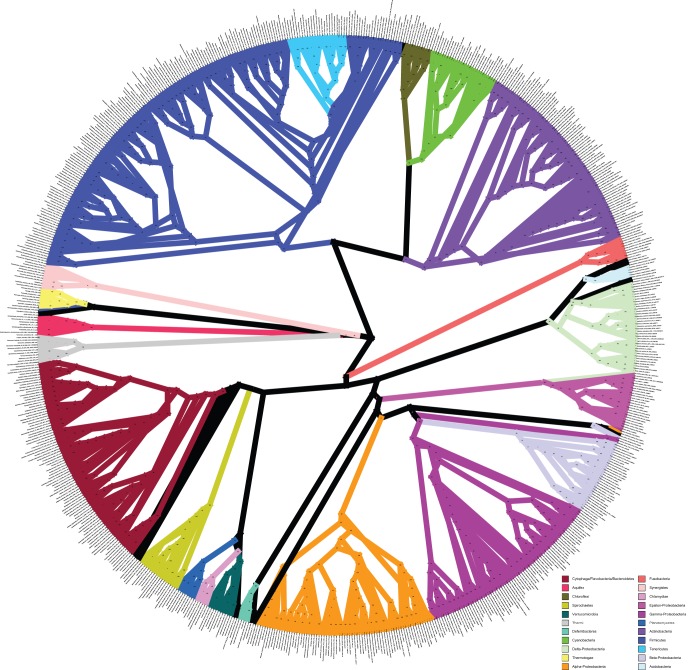
Concatenated Maximum Likelihood tree. Phylogenetic tree inferred from a concatenated, partitioned alignment of 24 genes using RAxML. The branches of phyla with at least 5 representatives are colored, other lineages are all drawn with black lines. Support values are calculated from 100 rapid bootstrap replicates. This representation is a radial cladogram, in which branch length is not proportional to time, and some branches may be elongated so that the names of the taxa appear on the circumference of the circle. The original version of this figure is available in the Supporting Information: Figure S7.

**Figure 2:**
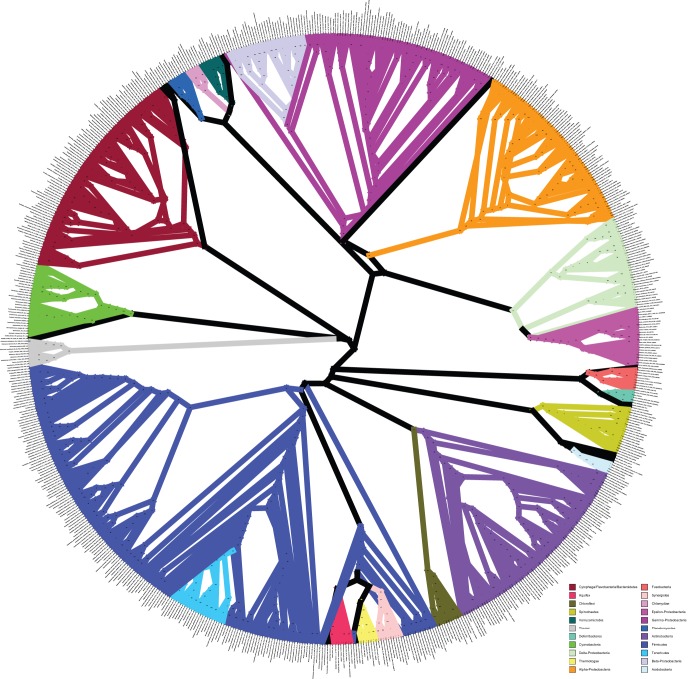
16S rRNA Maximum Likelihood tree. Phylogenetic tree inferred from an alignment of the 16S rRNA gene using RAxML. The branches of phyla with at least 5 representatives are colored, other lineages are all drawn with black lines. Support values are calculated from 100 bootstrap replicates. This representation is a radial cladogram, in which branch length is not proportional to time, and some branches may be elongated so that the names of the taxa appear on the circumference of the circle. The original version of this figure is available in the Supporting Information: Figure S8.

**Figure 3:**
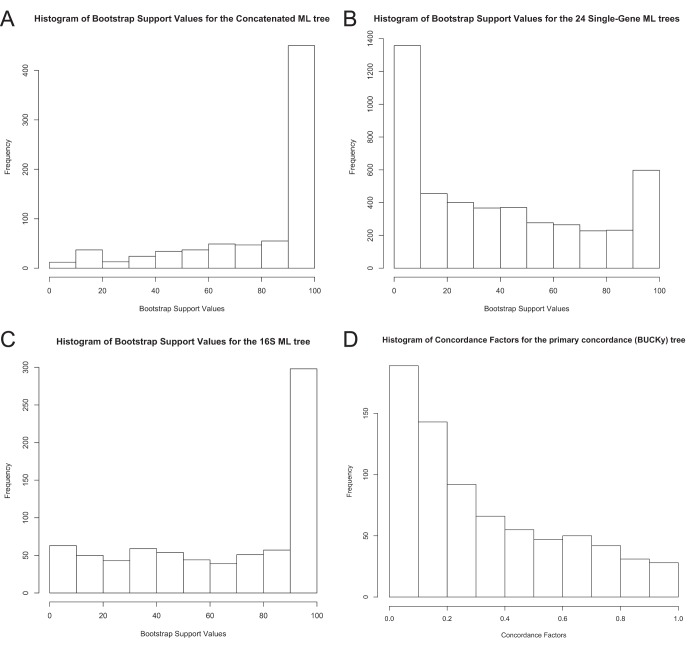
Frequencies of support values observed in phylogenetic trees. Histograms showing the frequency of support (bootstrap or concordance factor) values in the (A) best ML tree inferred from a concatenated alignment of 24 genes, (B) best ML trees for all of the 24 individual genes, (C) best ML tree inferred from the 16S rRNA gene, and (D) primary concordance (“BUCKy”) tree.

The tree in Figure 1 is the result of the full ML search using the concatenated alignment with clade support values obtained from the rapid bootstrap replicates (also see Figure S1 for a horizontal depiction of this tree, which some readers will find easier to interpret, or Datafile S1 for a text representation of the tree in the Newick format suitable for most tree-viewing applications). It recovers most of the clades that are expected based on the 16S rRNA phylogeny (Figure 2, Figure S2, and Datafile S2), but offers greater clade support overall (Figure 3), and increased resolution among phyla. There are some notable differences between the 16S rRNA tree and the concatenated ML tree, which will be discussed below.

### BUCKy

BUCKy was designed to take as input the posterior distribution of tree topologies generated by MrBayes for each gene. Because the single-gene Bayesian analyses could not be run to convergence due to computational limitations, we used an alternative approach suggested by [40]. The rapid bootstrapping algorithm implemented in RAxML was used to generate 1000 bootstrap replicates for each of the 24 genes. These were used as input for BUCKy. BUCKy crashed when given 1000 bootstrap replicates, but ran with 500 replicates, and because the rapid bootstrapping had converged in all cases by 500 replicates, we used that number as input for BUCKy. Because BUCKy typically takes MrBayes output as input for analysis, a custom Perl script was required to modify the RAxML output to serve this purpose (Datafile 7). We generated a primary concordance (BUCKy) tree using the single-gene RAxML bootstrap replicate trees as input.

The trees produced using alpha = 10, 50, and 100 were identical, both in topology and the values of the concordance factors at every node. Because we know that there is considerable uncertainty in the single-gene trees, we chose to use the primary concordance tree that was generated using a larger prior than the default of 1. The RF distance between the alpha = 1 and alpha = 10 primary concordance trees was (72/4.76%) and the average concordance factor value was (0.360) in the alpha = 1 tree, versus (0.368) in the alpha = 10 tree. The main (minor) difference between the two trees is the degree of resolution: the alpha = 1 tree has 1508 splits and the alpha = 10 tree has 1512 splits. For reference, a fully resolved tree has 1522 splits.

**Figure 4:**
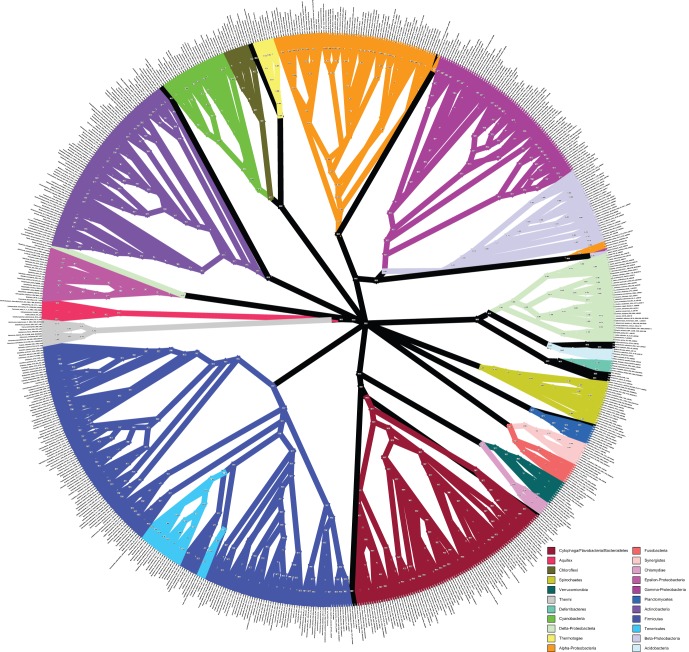
BUCKy tree. Primary concordance (“BUCKy”) tree constructed using Bayesian Concordance Analysis of RAxML bootstrap replicates for each of the 24 phylogenetic marker genes. Values at the nodes are concordance factors. The branches of phyla with at least 5 representatives are colored, other lineages are all drawn with black lines. This representation is a radial cladogram, in which branch length is not proportional to time, and some branches may be elongated so that the names of the taxa appear on the circumference of the circle. The original version of this figure is available in the Supporting Information: Figure S9.

**Figure 5:**
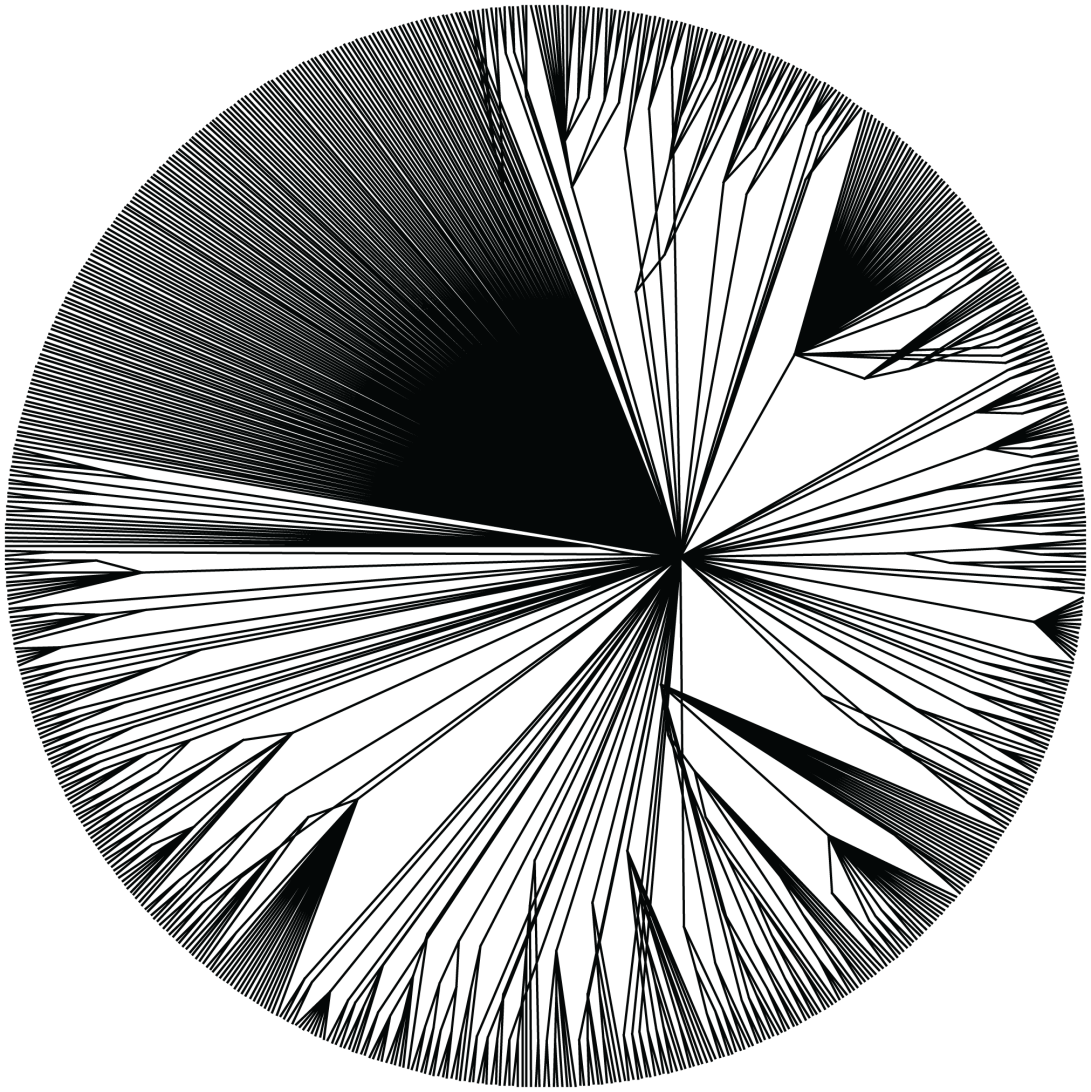
A majority-rule consensus tree calculated from the bootstrap replicates of one of the 24 genes. Majority-rule consensus tree computed from the bootstrap replicates for the 30S ribosomal protein S3. This gene has an alignment length of 180 sites, which is the average length for the 24 marker genes used in this study. This representation is a radial cladogram, in which branch length is not proportional to time, and some branches may be elongated so that the taxa appear on the circumference of the circle. The original version of this figure is available in the Supporting Information: Figure S10.

**Figure 6:**
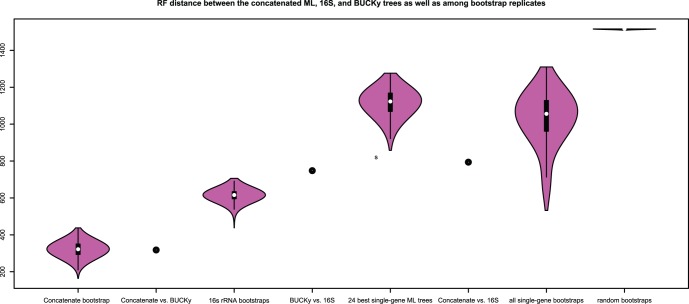
Robinson-Foulds distances between trees. Violin plot depicting the distribution of Robinson-Foulds (RF) distance measures among all pairwise comparisons between bootstrap replicates and between the best 24 single-gene maximum likelihood trees produced by RAxML. Points are plotted on the graph to show the RF values for pairwise comparisons between the concatenated ML tree vs. the BUCKy tree, between the BUCKy tree and the 16S rRNA tree, and between the concatenated ML tree and the 16S rRNA tree.

**Figure 7:**
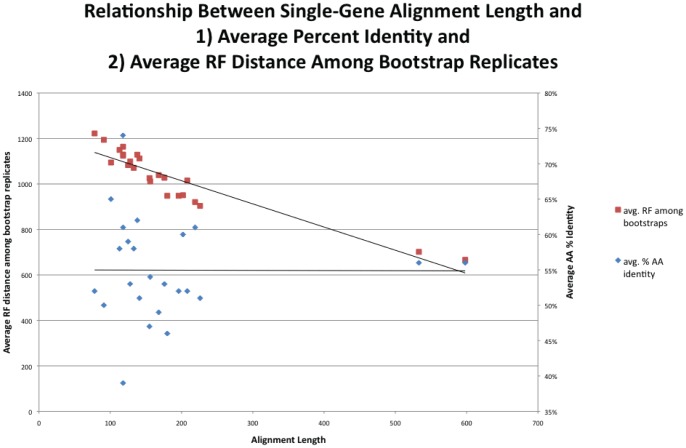
Correlation of gene alignment length and average amino acid identity with variance among bootstrap replicates. Scatter plot showing the negative correlation of alignment length vs. average Robinson-Foulds (RF) distance among bootstrap replicates for each of the 24 genes. The average percent identity of each alignment is not correlated with the average RF distance among bootstrap replicates for each of the 24 genes.

The BUCKy tree (Figure 4, Figure S5, and Datafile S5) showed a lack of resolution of the relationships deep in the bacteria, as evidenced by the fact that the relationships among phyla are represented by a large polytomy. It also exhibited, for the most part, very low concordance factors (Figure 3D), relative to the bootstrap support values obtained for the concatenated alignment. Concordance factors represent an estimate of the proportion of gene trees that have a particular clade. The method by which the primary concordance tree is computed is to rank clades based on their concordance factors, from high to low, and then assemble the tree such that none of the clades present conflict with clades having a higher concordance factor [44]. While it is possible that the 24 genes do have conflicting evolutionary histories, we conclude that the low concordance factors observed in our BUCKy tree, in particular at the nodes that are well-supported by the concatenated analysis, are due instead to the lack of phylogenetic signal of individual genes. It is known (Cecile Ane, pers. comm.) that BUCKy will underestimate concordance factors when there are large numbers of taxa and poorly resolved gene trees, but that the inference of topology is robust to these conditions. Examination of the majority-rule consensus trees for all of the single gene analyses (see Figure 5 for an example) reveals that there is very little resolution beyond grouping a few species of the same genera. A marjority-rule consensus tree of the 24 best ML single-gene trees reveals the same lack of resolution (Figure S6 and Datafile S6). This lack of resolution explains the low concordance factors, and suggests that they are low, not because there is a lot of conflicting signal between the genes, but that the signal for each gene is very weak. Further evidence for this interpretation comes from a comparison of the Robinson-Foulds distance metric computed for all pairwise comparisons of bootstrap replicate trees for each gene (Figure 6). The average RF distance among these bootstrap replicates is closer to the average distance among 100 random trees than it is to the average RF distance among bootstrap replicates of the concatenated alignment. There is also a significant negative correlation between the length of the single-gene alignments and the RF distance among bootstrap replicates, suggesting that it is a reduction in information content of the genes that is leading to an increase in the variance of the phylogenetic inference. The average percent identity, however, is similar for all genes, including the two longest genes, which also are the only two protein-encoding genes included that are not known to operate in the ribosome (see Figure 7). Unfortunately, there is no way to increase the information content of a single gene. Nevertheless, the BUCKy tree is remarkably similar in topology to the concatenated ML tree. The RF distance between the BUCKy tree and the concatenated ML tree is 318, and the average RF among bootstrap replicates for the concatenated ML tree is 322. While it has been suggested that concatenating single-gene alignments can result in the emergence of phylogenetic signal that is missing from single genes [34], our results suggest that BUCKy can effectively be used to extract this signal without resorting to concatenation. Unfortunately, the preponderance of low concordance factors makes tree interpretation and comparisons difficult, as it is unclear as to what should be considered a “well-supported” clade. Also, the BUCKy tree does not include branch lengths, limiting its utility for some applications.

### Placement of Interesting Taxa in the Tree

For many organisms, especially those that have been selected for genome sequencing due to their phylogenetic novelty or are otherwise from relatively under-sampled clades, the best estimate of their phylogenetic history has been derived from their position in the 16S rRNA gene phylogeny. Our results show that there is a great deal of congruence/agreement between the phylogenetic trees obtained by analysis of the 16S rRNA gene and that of the 24 genes used here, especially in terms of recovering known phyla (see Figures 1 and 2). However, there are a few noteworthy differences, including an increased resolution of the relationships among phyla in the concatenated ML tree, which is not surprising, given the additional data. Because most relationships between bacterial phyla have historically been entirely unresolved, it was our thought that any move towards resolution was noteworthy, and we therefore used a fairly permissive threshold to define an increase in resolution. We define this increase in phylogenetic resolution as the appearance of at least moderately well-supported (>50% bootstrap) clades in the concatenated ML tree that are not found (with at least 50% bootstrap support) in the 16S rRNA tree. For the purposes of this discussion, we will only be comparing the 16S rRNA tree to the concatenated ML tree. The BUCKy tree will be mentioned only when it is in conflict (with a high concordance factor) with the concatenated ML tree.

#### Planctomycetes

The placement of the Planctomycetes has been somewhat controversial [74], [75], and in the 16S rRNA tree, they are part of a basal polytomy in the bacteria. In our multi-gene phylogeny, they are sister to the *Chlamydiae/Verrumicrobia* group with 86% bootstrap support.

#### Thermodesulfobium narugense and coprothermobacter proteolyticus

The published description of *Thermodesulfobium narugense* [76] describes it as a representative novel lineage of sulfate-reducing thermophiles, most closely related to the candidate phylum OP9, but less than 81% similar to any 16S rRNA sequences (from either isolate or environmental clones). In that study, the authors generated trees based on two genes involved in sulfate reduction, *dsr*AB and *aps*A. These two genes are found in only a few bacterial and archaeal clades, so they were not useful for confirming the phylogenetic placement of *T. narugense*, but they do have conflicting topologies that the authors state may be due to HGT of the sulfate reduction genes (which has been proposed to be frequent). It appeared to have a “distinctive” *dsr*A gene, while its *Aps*A gene appeared to have been most closely related to that of *D. hydrogenovorans* (a delta-proteobacterium). In our 16S rRNA tree of all sequenced genomes, it is a part of a basal polytomy of the bacteria. In the concatenated ML tree, it is sister to *Dictyglomus* (boostrap support = 60%) and those two are sister to the *Thermotogae*+*Coprothermobacter* with moderate (53%) bootstrap support. This corroborated a recently published anlysis of 44 orthologous protein sequences, which demonstrated a sister relationship between *Dictyglomus* and *Thermotogea*, but did not include *Thermodesulfobium* [77]. The clade consisting of (*Thermodesulfobium*+*Dictyglomus*) and (*Thermotogae*+*Coprothermobacter*) have a strongly-supported (bootstrap = 83%) sister relationship to a clade containing the *Deinococcus*-*Thermus* group and the *Aquificaceae*.

#### Hippea

*Hippea maritima* is a thermophilic, sulfur-reducing bacterium that was isolated from shallow submarine hot vents [78]. It was previously placed in the family *Desulfurellaceae* based on its 16S rRNA sequence similarity (89.6%) to *Desulfurella multipotens* and placement as sister to the *Desulfurella* clade of the delta-proteobacteria [79]. However, our concatenated ML tree shows strong (bootstrap = 100%) support for its placement as a lineage basal to the rest of the epsilon-proteobacteria, thus we propose that it be reclassified as an epsilon-proteobacteria.

#### Acidithiobacillus

In all trees presented here, the gamma-proteobacteria is a paraphyletic group. In the concatenated ML tree, with the exception of Candidatus *Carsonella rudii*, which is on a long, unstable branch (see discussion of LBA above), the *Acidithiobacillus* species are the only gamma-protobacterial taxa that are not contained within a well-supported (bootstrap support = 70%), monophyletic clade. The two *Acidithiobacillus* species instead represent a distinct lineage, basal to the clade (bootstrap = 48%) containing the beta+alpha-proteobacteria. In the BUCKy tree, they are the basal lineage of the beta-proteobacterial clade. The placement of this group has historically been problematic [80], and we propose that it be classified as a lineage, distinct from the gammaproteobacteria, called the eta-proteobacteria.

#### Thermodesulfobacterium

*Thermodesulfobacterium* has been proposed to represent a deeply-diverging division-level lineage [81], [82]. Lateral transfer of the genes involved in dissimilatory sulfite reduction has been proposed because the *dsr*AB genes of *Thermodesulfobacterium* species are most closely related to those of the delta-proteobacteria. The concatenated ML tree provides strong support (bootstrap = 100%) for the sister relationship of *Thermodesulfobacterium commune*+*Thermodesulfatator indicus* to the *Desulfovibrionales*, a family within the delta-proteobacteria. This result, along with the fact that these organisms have a similar metabolism to other *Desulfovibrionales* [81], [83], suggests that the topological discordance between the 16S rRNA tree and the *drs*AB tree that was interpreted in [82] as evidence for lateral transfer of the *dsr*AB gene, is in fact due to an incorrect placement of *T. commune* in the 16S rRNA tree.

#### Acidobacteria, nitrospirae, and poribacteria

In the concatenated ML tree, there is strong support for the sister relationship of the *Acidobacteria* and the *Nitrospirae* (bootstrap = 89%). The strongly-supported *Nitrospirae* clade (bootstrap = 95%) contains *Thermodesulfovibrio yellowstonii*, Candidatus *Nitrospira defluvii*, and Candidatus *Poribacteria.* These two clades (*Acidobacteria+Nitrospira*) are, with moderate support (bootstrap = 55%), sister to the delta-proteobacteria. The inclusion of Candidatus *Poribacteria* in the *Nitrospirae* clade is in conflict with its placement in the 16S rRNA tree. In the 16S rRNA tree, *Poribacteria* is at the base of the *Planctomycetes* (bootstrap = 43%), and that clade is sister to the *Verrucomicrobia*+ *Chlamydia* clade (bootstrap = 54%). This “*Poribacteria*” genome was sequenced using a single-cell whole genome amplification (WGA) approach [84]. Contamination is a concern with this approach, and the authors took care to convince themselves that there was no contamination (other than from *Delftia*, which is a common beta-proteobacterial reagent contaminant) from other genomes in their sequence. Their evidence for lack of contamination was 1) a single copy of the 16SrRNA gene, 2) single copies of 29 of 55 known single-copy genes [85], and 3) a unimodal %GC distribution of the reads. The conflict between the 16S rRNA and concatenated ML phylogenies may be due to reasons other than contamination in the WGA product in cases like this. Nevertheless, we suggest that an additional check for contamination should be the phylogenetic analysis of other phylogenetic marker genes found in the genome sequence.

#### TM7 single-cell isolates

There are two TM7 genomes that were sequenced with the WGA approach. Using the 16S rRNA sequence from the WGA data, these two are sister taxa with bootstrap support of 100%, and they are placed, with weak support, as sister to the *Chloroflexi*. In the concatenated ML tree, they are not sister taxa. TM7c is placed with weak support (bootstrap = 38%) as sister to *Anaerolina thermophilia,* within the *Chloroflexi*. The other candidate division TM7 genomosp. GTL1 isolate is placed with very weak support within a clade containing three insect symbionts, basal to the gamma+beta-proteobacterial clade. This placement is possibly the result of an LBA artifact. While the true placement of these two genomes is unclear based on the concatenated ML tree, it is noteworthy that they do not have the strongly-supported sister relationship that we see in the 16S rRNA tree.

### Summary/Conclusion

We were interested in generating a single, fully resolved phylogenetic tree to be used in comparative analyses. We chose 24 highly-conserved, single-copy genes to be used for phylogenetic analysis. We wanted to use an approach that avoids some of the (unrealistic) assumptions about how genes evolve, especially in microbes. In particular, we wanted to use an approach that does not assume that every gene shares a single phylogenetic history, i.e., a *supertree* approach. A review of available methods (as well practical difficulties with Bayesian approaches) led us to believe that the best strategy, given our data, was to use Bayesian Concordance Analysis (as implemented in BUCKy) with RAxML bootstrap replicates as input. We were interested in how a tree produced by BUCKy in this way compared to a ML analysis of a concatenated alignment. Our analyses including both bacteria and archaea revealed obvious long branch attraction artifacts within the bacteria. We therefore opted to focus all of our tree (method) comparisons on trees that contained only bacterial taxa, in which the long branch attraction artifact was ameliorated.

For the Bayesian Concordance Analysis, we first attempted to use Bayesian phylogenetic inference, but current methods were unable to mix and converge on our data after many months of CPU time. Instead, we opted to employ a maximum likelihood approach using RAxML. We used two approaches to combine the data from the 24 phylogenetic marker genes. The first approach was to concatenate alignments of all 24 genes and run a single ML analysis, with bootstrap replicates. The second approach was to generate many bootstrap replicates from an alignment of each gene, and then combine the information from the single gene trees using BUCKy. We also performed ML analysis of an alignment of 16S rRNA sequences for each of the taxa included in the other two trees. This 16S rRNA tree was used as a standard to which to compare the phylogenetic placement of particular taxa in our concatenated ML and BUCKy trees.

The concatenated ML tree and the BUCKy tree were more similar to each other than either was to the 16S rRNA tree. There are differences between the arrangements of taxa in all three trees, but most of the major bacterial groupings (phylum-level) were shared among all three. The ML tree based on the concatenated alignment was better-supported overall than the 16S rRNA tree (Figure 3) and the topological variance among bootstrap replicates inferred from the concatenated alignment was lower than among the 16S rRNA bootstrap replicates.

As has been observed in previous, similar simulations, a Bayesian approach to the reconstruction of phylogenies of this size is currently computationally infeasible [40]. The computation time for the maximum likelihood analyses run here was more reasonable, especially when using cluster or cloud computing. The run time for the concatenated alignment was approximately 2 weeks (distributed over 8 cores), and the single-gene analyses took approximately 1 day per gene (again, distributed over 8 cores). The runtime for BUCKy to produce the primary concordance tree was approximately 4 hours.

From among the trees we generated during this process, we would choose the concatenated ML tree as our working representation of the relationships among bacterial genomes for 4 reasons: 1) it produced a fully resolved tree, which is essential for many of the downstream analyses that we intend to do; 2) it provides an estimate of branch lengths, which is not only generally informative, but also essential for many downstream analyses; 3) it is accompanied by support values that are meaningful, if for no other reason, than the community at large is accustomed to an intuitive interpretation of bootstrap support values, where as the concordance factors produced by BUCKy, when this large number of taxa are analyzed, are currently difficult to interpret, (Cecile Ane, pers. comm.); and 4) because it is much simpler and less time-consuming to run than any of the other methods we tested here.

All alignments and trees produced in this study are freely available via Figshare (www.figshare.com).

## Supporting Information

Figure S1 **Maximum likelihood tree of bacteria from concatenated alignment (horizontal format).** Phylogenetic tree of 761 bacterial taxa, inferred from a concatenated, partitioned alignment of 24 genes using RAxML. The branches of phyla with at least 5 representatives are colored, other lineages are all drawn with black lines. Support values are calculated from 100 rapid bootstrap replicates. This is the same tree as Figure 1 from the main text, shown in a horizontal format.
(PDF)

Figure S2 **Maximum likelihood tree of bacteria from 16S rRNA genes (horizontal format).** Phylogenetic tree of 761 bacterial taxa, inferred from an alignment of the 16S rRNA gene using RAxML. The branches of phyla with at least 5 representatives are colored, other lineages are all drawn with black lines. Support values are calculated from 100 bootstrap replicates. This is the same tree as Figure 2 from the main text, shown in a horizontal format.
(PDF)

Figure S3 **FastTree of all bacteria and archaea analyzed (2966 taxa) from 38 concatenated genes.** Phylogenetic tree of 2966 bacterial and archaeal taxa, inferred from a concatenated alignment of 38 marker genes (Wu et al, in prep) using FastTree.
(SVG)

Figure S4 **Maximum likelihood tree of bacteria and archaea from concatenated alignment.** Phylogenetic tree of 841 bacterial and archaeal taxa, inferred from an alignment of 24 marker genes using RAxML. Support values are calculated from 100 bootstrap replicates.
(PDF)

Figure S5 **BUCKy tree (horizontal format).** Primary concordance (“BUCKy”) tree constructed using Bayesian Concordance Analysis of RAxML bootstrap replicates for each of the 24 phylogenetic marker genes. Values at the nodes are concordance factors. The branches of phyla with at least 5 representatives are colored, other lineages are all drawn with black lines. This is the same tree as Figure 4 from the main text, shown in a horizontal format.
(PDF)

Figure S6 **50% majority rule consensus computed from the 24 best ML single-gene trees generated by RAxML.**
(PDF)

Figure S7 **Concatenated Maximum Likelihood tree.** Phylogenetic tree inferred from a concatenated, partitioned alignment of 24 genes using RAxML. The branches of phyla with at least 5 representatives are colored, other lineages are all drawn with black lines. Support values are calculated from 100 rapid bootstrap replicates. This representation is a radial cladogram, in which branch length is not proportional to time, and some branches may be elongated so that the names of the taxa appear on the circumference of the circle.
(EPS)

Figure S8 **16S rRNA Maximum Likelihood tree.** Phylogenetic tree inferred from an alignment of the 16S rRNA gene using RAxML. The branches of phyla with at least 5 representatives are colored, other lineages are all drawn with black lines. Support values are calculated from 100 bootstrap replicates. This representation is a radial cladogram, in which branch length is not proportional to time, and some branches may be elongated so that the names of the taxa appear on the circumference of the circle.
(EPS)

Figure S9 **BUCKy tree.** Primary concordance (“BUCKy”) tree constructed using Bayesian Concordance Analysis of RAxML bootstrap replicates for each of the 24 phylogenetic marker genes. Values at the nodes are concordance factors. The branches of phyla with at least 5 representatives are colored, other lineages are all drawn with black lines. This representation is a radial cladogram, in which branch length is not proportional to time, and some branches may be elongated so that the names of the taxa appear on the circumference of the circle.
(EPS)

Figure S10 **A majority-rule consensus tree calculated from the bootstrap replicates of one of the 24 genes.** Majority-rule consensus tree computed from the bootstrap replicates for the 30S ribosomal protein S3. This gene has an alignment length of 180 sites, which is the average length for the 24 marker genes used in this study. This representation is a radial cladogram, in which branch length is not proportional to time, and some branches may be elongated so that the taxa appear on the circumference of the circle.
(EPS)

Table S1 **Organisms used in this study.** List of organisms used in this study, with (when available) NCBI accession numbers and IMG taxon ID numbers.
(XLS)

Datafile S1 **Maximum likelihood tree of bacteria from concatenated alignment (Newick format).** Phylogenetic tree of 761 bacterial taxa, inferred from a concatenated, partitioned alignment of 24 genes using RAxML. Support values are calculated from 100 rapid bootstrap replicates. This is the same tree as Figure 1 from the main text, shown in the Newick format, suitable for use in most tree-viewing programs.
(TXT)

Datafile S2 **Maximum likelihood tree of bacteria from 16S rRNA genes (Newick format).** Phylogenetic tree of 761 bacterial taxa, inferred from an alignment of the 16S rRNA gene using RAxML. Support values are calculated from 100 bootstrap replicates. This is the same tree as Figure 2 from the main text, shown in the Newick format, suitable for use in most tree-viewing programs.
(TXT)

Datafile S3 **FastTree of all bacteria and archaea analyzed (2966 taxa) from 38 concatenated genes (Newick format).** Phylogenetic tree of 2966 bacterial and archaeal taxa, inferred from a concatenated alignment of 38 marker genes (Wu *et al.*, in prep) using FastTree. This Newick format is suitable for use in most tree-viewing programs.
(TXT)

Datafile S4 **Maximum likelihood tree of bacteria and archaea from concatenated alignment (Newick format).** Phylogenetic tree of 841 bacterial and archaeal taxa, inferred from an alignment of 24 marker genes using RAxML. Support values are calculated from 100 bootstrap replicates. This Newick format is suitable for use in most tree-viewing programs.
(TXT)

Datafile S5 **BUCKy tree (Newick format).** Primary concordance (“BUCKy”) tree constructed using Bayesian Concordance Analysis of RAxML bootstrap replicates for each of the 24 phylogenetic marker genes. Values at the nodes are concordance factors. This is the same tree as Figure 4 from the main text, shown in the Newick format, suitable for use in most tree-viewing programs.
(TXT)

Datafile S6 **50% majority rule consensus computed from the 24 best ML single-gene trees generated by RAxML (Newick format).** This is the same tree as Figure S6, shown in the Newick format, suitable for use in most tree-viewing programs.
(TXT)

Datafile S7 **Perl script that can be used (upon installation of R) to convert RAaML bootstrap replicates into a format that can be used as input for BUCKy.**
(TXT)
