# Supplementary figures and images for: Phylogeny of Bacterial and Archaeal Genomes Using Conserved Genes: Supertrees and Supermatrices
Source: PLoS One. 2013 Apr 25;8(4):e62510. doi: 10.1371/journal.pone.0062510 (PMC3636077; doi:10.1371/journal.pone.0062510)

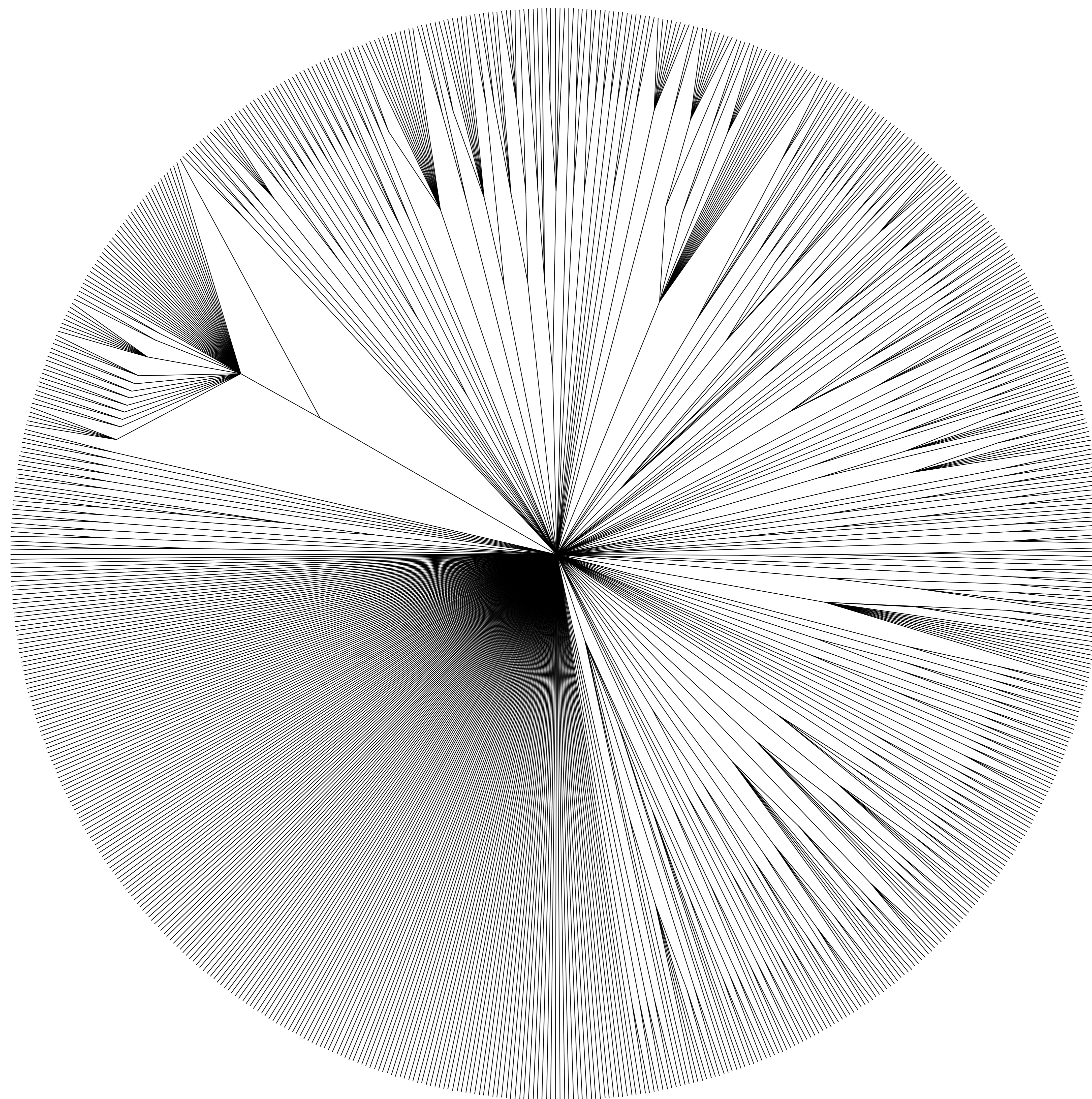

Supplement: Figure S6 — 50% majority rule consensus computed from the 24 best ML single-gene trees generated by RAxML. (PDF) [file pone.0062510.s006.pdf]
