# Supplementary material for: Phylogeny of Bacterial and Archaeal Genomes Using Conserved Genes: Supertrees and Supermatrices
Source: PLoS One. 2013 Apr 25;8(4):e62510. doi: 10.1371/journal.pone.0062510 (PMC3636077; doi:10.1371/journal.pone.0062510)

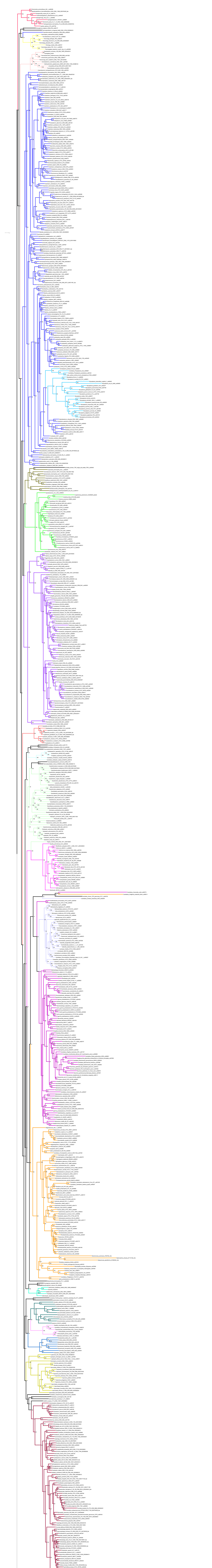

Supplement: Figure S1 — Maximum likelihood tree of bacteria from concatenated alignment (horizontal format). Phylogenetic tree of 761 bacterial taxa, inferred from a concatenated, partitioned alignment of 24 genes using RAxML. The branches of phyla with at least 5 representatives are colored, other lineages are all drawn with black lines. Support values are calculated from 100 rapid bootstrap replicates. This is the same tree as Figure 1 from the main text, shown in a horizontal format. (PDF) [file pone.0062510.s001.pdf]
