# Supplementary material for: Phylogeny of Bacterial and Archaeal Genomes Using Conserved Genes: Supertrees and Supermatrices
Source: PLoS One. 2013 Apr 25;8(4):e62510. doi: 10.1371/journal.pone.0062510 (PMC3636077; doi:10.1371/journal.pone.0062510)

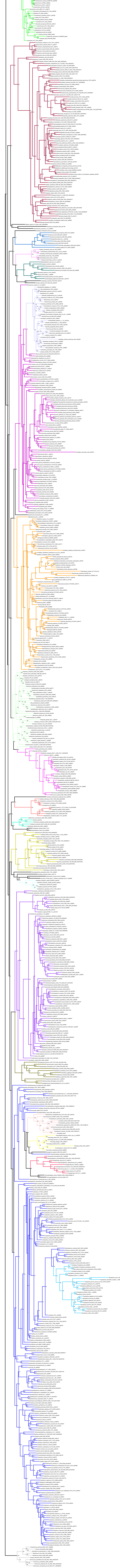

Supplement: Figure S2 — Maximum likelihood tree of bacteria from 16S rRNA genes (horizontal format). Phylogenetic tree of 761 bacterial taxa, inferred from an alignment of the 16S rRNA gene using RAxML. The branches of phyla with at least 5 representatives are colored, other lineages are all drawn with black lines. Support values are calculated from 100 bootstrap replicates. This is the same tree as Figure 2 from the main text, shown in a horizontal format. (PDF) [file pone.0062510.s002.pdf]
