# Supplementary material for: Phylogeny of Bacterial and Archaeal Genomes Using Conserved Genes: Supertrees and Supermatrices
Source: PLoS One. 2013 Apr 25;8(4):e62510. doi: 10.1371/journal.pone.0062510 (PMC3636077; doi:10.1371/journal.pone.0062510)

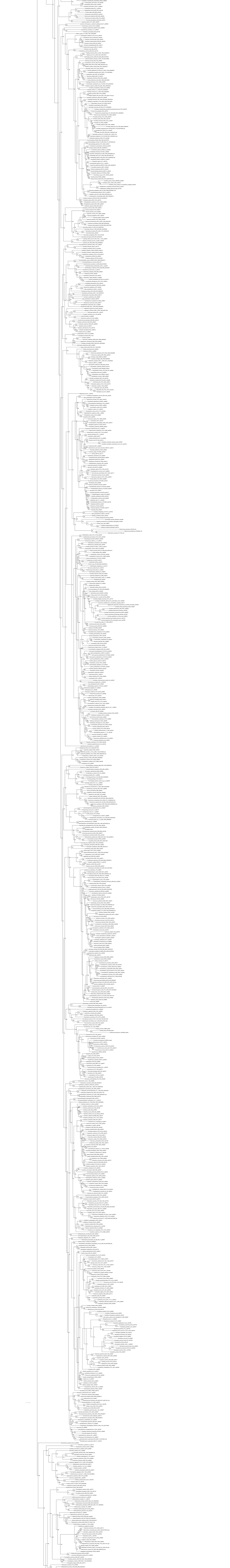

Supplement: Figure S4 — Maximum likelihood tree of bacteria and archaea from concatenated alignment. Phylogenetic tree of 841 bacterial and archaeal taxa, inferred from an alignment of 24 marker genes using RAxML. Support values are calculated from 100 bootstrap replicates. (PDF) [file pone.0062510.s004.pdf]
