# Supplementary material for: Phylogeny of Bacterial and Archaeal Genomes Using Conserved Genes: Supertrees and Supermatrices
Source: PLoS One. 2013 Apr 25;8(4):e62510. doi: 10.1371/journal.pone.0062510 (PMC3636077; doi:10.1371/journal.pone.0062510)

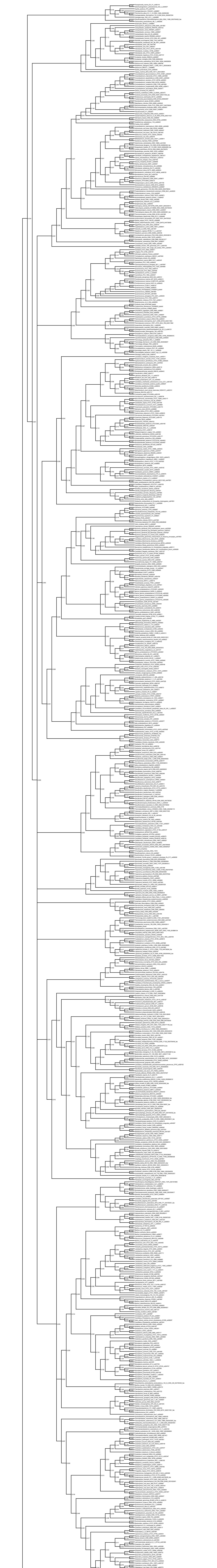

Supplement: Figure S5 — BUCKy tree (horizontal format). Primary concordance (“BUCKy”) tree constructed using Bayesian Concordance Analysis of RAxML bootstrap replicates for each of the 24 phylogenetic marker genes. Values at the nodes are concordance factors. The branches of phyla with at least 5 representatives are colored, other lineages are all drawn with black lines. This is the same tree as Figure 4 from the main text, shown in a horizontal format. (PDF) [file pone.0062510.s005.pdf]
